# The Resolved Mutual Information Function as a Structural Fingerprint of Biomolecular Sequences for Interpretable Machine Learning Classifiers

**DOI:** 10.3390/e23101357

**Published:** 2021-10-17

**Authors:** Katrin Sophie Bohnsack, Marika Kaden, Julia Abel, Sascha Saralajew, Thomas Villmann

**Affiliations:** 1 Saxon Institute for Computational Intelligence and Machine Learning, University of Applied Sciences Mittweida, 09648 Mittweida, Germany; kaden1@hs-mittweida.de (M.K.); abel@hs-mittweida.de (J.A.); 2 Bosch Center for Artificial Intelligence, 71272 Renningen, Germany; sascha.saralajew@de.bosch.com

**Keywords:** mutual information, sequence analysis, classification, machine learning, interpretable models

## Abstract

In the present article we propose the application of variants of the mutual information function as characteristic fingerprints of biomolecular sequences for classification analysis. In particular, we consider the resolved mutual information functions based on Shannon-, Rényi-, and Tsallis-entropy. In combination with interpretable machine learning classifier models based on generalized learning vector quantization, a powerful methodology for sequence classification is achieved which allows substantial knowledge extraction in addition to the high classification ability due to the model-inherent robustness. Any potential (slightly) inferior performance of the used classifier is compensated by the additional knowledge provided by interpretable models. This knowledge may assist the user in the analysis and understanding of the used data and considered task. After theoretical justification of the concepts, we demonstrate the approach for various example data sets covering different areas in biomolecular sequence analysis.

## 1. Introduction

The accumulation of information based on physical organization processes like structure generation and self-organization belongs to the key aspects of living systems [1,2,3,4]. Thus, information theoretic concepts play an important role in sequence analysis for understanding biomolecular entities like RNA, DNA, and proteins to explain biological systems [5,6,7]. In case of DNA/RNA, the biomolecular information is coded by the nucleotide sequence, particularly their sequence element’s frequencies, correlations, and other topological features. The extensive influence of information theoretic concepts and applications in the fields of computational, molecular, and systems biology is captured in various reviews [8,9,10,11].

The study of sequences in consideration of their biological properties is still crucial for such diverse applications as drug design, phylogenetic analyses, prediction of molecular interactions, identification of polymorphisms or definition of pathogenic mutations [12,13,14,15]. With the availability of powerful machine learning methods like deep and convolutional networks [16,17], and support vector machines [18] as well as the supporting hardware (graphic processing units—GPU), self-learning procedures have entered (and revolutionized) many of these areas of biomolecular research [19,20,21,22,23]. Although these models provide promising performance by automated training and outperform many statistical approaches, the disadvantage is their general “black-box” behavior, i.e., the model decisions are usually hardly interpretable. Thus, explanations are at least difficult to give and usually require additional tools [24]. However, current focus is given to develop interpretable machine learning models instead [25,26]. According to [27], interpretable models are *designed* to be interpretable in contrast to explainable methods, which can be comprehended post-hoc by experts in the field using additional tools and elaborate considerations. Generally, interpretability increases the trustworthiness of the machine learning method and hence contributes to making them transparent for the applicants. However, interpretable models require meaningful features describing the objects to be considered, ideally taking domain knowledge into account.

It is precisely this identification or rather the design of problem-adequate features that is the subject of research in the field of alignment-free sequence comparison in computational biology. By overcoming some of the major disadvantages of alignments, such as strong evolutionary assumptions [28], high computational costs [29] as well as non-numerical sequence representation, alignment-free methods evolved as a true alternative for quantifying sequence (dis-)similarity [30]. At present, respective methods are used in the domains of phylogenetics [31,32,33], (meta-)genomics [34,35], database similarity search [36], or next-generation sequencing data analyses [37,38,39].

In particular, information theoretic and statistical quantities provide a natural way to generate unique signatures or fingerprints of molecular sequence data by considering the distribution of nucleotides as elements of sequence as well as their statistical correlations [40,41,42]. Long-range correlations in sequences are well-known and intensively studied in alignment-free sequence comparison [43,44,45,46]. A promising statistical descriptor approach for sequences is the concept of natural vectors. It considers the moments of the distribution of the nucleotides in a sequence as the determining quantities to characterize the molecular sequence [47]. The equality of all statistical moments of two sequences implies the equality of statistical distributions and, hence, can be seen as an equivalence relation. Natural vectors were successfully applied for DNA-analysis, virus, and protein sequence classification [48,49].

The use of so-called mutual information functions (MIFs) as an alternative to correlation profiles as sequence descriptors was first investigated in [50]. This idea was reconsidered in [51] and renewed in [52,53]. In bioinformatics, this concept was established as average mutual information profile (AMI-profile) and proposed to serve as a genomic signature [54]. Molecular descriptors based on the mutual information are considered in [55]. Further applications of MIF in computational biology involve its use for species identification from DNA sequences [56], for finding (species-independent) patterns that differ in coding and non-coding DNA [57] as well as for investigating co-variation of mutations in protein sequences [58]. To make the idea accessible to a larger audience, a user interface program for MIF calculation is provided in [59] and more applications are reviewed in [60].

The mutual information is known as a similarity measure between distributions, which originally is based on the Shannon entropy [61]. It implicitly takes all correlation moments of the distributions for the comparison into account. Popular alternatives to the standard mutual information are the Rényi and the Tsallis mutual information, which are based on the Rényi and the Tsallis entropy, respectively [62,63,64,65]. The numerical estimations of these mutual information variants seem to be more robust than the estimations for the Shannon original [66].

These mutual information concepts can be used to generate information theoretic features for sequence analysis: Rényi entropic profiles were considered for DNA classification problems based on chaos game representation [67,68]. Molecular descriptors based on the Rényi entropy were investigated in [69], whereas long range correlation using Tsallis mutual information was considered in [70]. However, to our best knowledge, MIF for these variants are not known so far.

Furthermore, in [71], it is criticized that the (Shannon) AMI profile, i.e., the MIF, suffers from an average effect of 16 kinds of base correlations. Therefore, the authors of this study proposed, based on the earlier work in [40], a partial information correlation.

This critic together with the above mentioned robustness observations for the Rényi and the Tsallis entropy estimators motivated our investigations: first, we introduce a resolved variant of the Shannon-based MIF as a more adequate information theoretic signature of molecular sequences reducing the average effects. Afterwards, we transfer this concept to both the Rényi and the Tsallis variants obtaining respective (resolved) mutual information functions. The resulting signature vectors serve as data descriptors for sequence classification problems to be tackled by machine learning methods. In this machine learning part, we focus on dissimilarity based and interpretable classifier models according to the above discussion about interpretability. Particularly, we apply a variant of learning vector quantization which delivers feature correlation information regarding the classification problem as an additional information beyond the classifier predicting performance [72]. Furthermore, this method is known to be robust and optimizing the class separating hypothesis margin [73].

The paper is structured as follows: first, we introduce variants of the mutual information functions for the Shannon-, the Rényi-, and the Tsallis-entropy and give theoretical justifications. Second, we describe the interpretable machine learning classifier based on learning vector quantization and show how knowledge about the decision process and the regarding data properties can be extracted. Thereafter, we apply this methodology for three biomolecular sequence data sets covering different application areas. For this purpose, we describe in detail the feature generation and the parameter setting. Furthermore, we show in the example for one data set how knowledge extraction from the trained classifier model is done to provide useful additional information. Concluding remarks and outlook for future work complete the paper.

## 2. Variants of Mutual Information Functions as Biomolecular Sequence Signatures

In the following section, we introduce the concept of variants of mutual information functions, which later serve as determining fingerprints of nucleotide sequences. These functions reflect structural characteristics and spatial relations within the sequences. For this purpose, we consider several types of mutual information regarding different entropy concepts. Thereby, we concentrate on those approaches, which are frequently used in machine learning. For a general overview of entropies, divergences, and mutual information, we refer to [74].

### 2.1. The Resolved Mutual Information Function Based on the Shannon Entropy

We consider the Shannon entropy

(1) $$H\left(X\right)=\int_{X}p\left(x\right)\cdot \log\left(\frac{1}{p\left(x\right)}\right)dx$$

of a random quantity X⊆X with the density measure $p\left(x\right)$ being the expectation value of the information $\log\left(\frac{1}{p\left(x\right)}\right)$. In the machine learning context here, we interpret *X* as a feature or object quantity. The maximum value of the entropy $H\left(X\right)$ is obtained for a uniform density $p\left(x\right)$ and, hence, $H\left(X\right)$ serves as a measure of uncertainty [75].

The corresponding divergence is the Kullback–Leibler-divergence

(2) $$D_{KL}\left(p\left(x\right)\|p\left(y\right)\right)=\int_{X}\int_{Y}p\left(x\right)\cdot \log\left(\frac{p\left(x\right)}{p\left(y\right)}\right)dydx$$

as dissimilarity measure between the densities $p\left(x\right)$ and $p\left(y\right)$ [61,76]. The corresponding mutual information is

(3) $$I\left(X,Y\right)=D_{KL}\left(p\left(x,y\right)\|p\left(x\right)\cdot p\left(y\right)\right)$$

quantifying the joint information of $p\left(x\right)$ and $p\left(y\right)$. Here, $p\left(x,y\right)$ is the joint density. Alternatively, the mutual information can be written as

(4) $$I\left(X,Y\right)=H\left(X\right)-H\left(X|Y\right)$$

using the conditional entropy $H\left(X|Y\right)$ which can be written as

(5) $$\begin{array}{ccc} H\left(X|Y\right) & = & H\left(X,Y\right)-H\left(Y\right)\\ & = & -\int_{X}\int_{Y}p\left(x,y\right)\cdot \log\left(\frac{p\left(x,y\right)}{p\left(y\right)}\right)dydx \end{array}$$

known as the chain rule of the entropies [61,76]. Equivalently, the mutual information can be formulated as the difference between the sum of the marginal entropies and the joint entropy, i.e.,

(6) $$I\left(X,Y\right)=H\left(X\right)+H\left(Y\right)-H\left(X,Y\right)$$

is valid.

We can rewrite the divergence formulation of the mutual information $I\left(X,Y\right)$ from Equation (3) as

$$I\left(X,Y\right)=\int_{X}F\left(x,Y\right)dx$$

where

(7) $$F\left(x,Y\right)=\int_{Y}p\left(x,y\right)\cdot \log\left(\frac{p\left(x,y\right)}{p\left(x\right)\cdot p\left(y\right)}\right)dy$$

describes a mutual information relation of a particular object (feature) *x* with respect to the random quantity *Y*. We denote $F\left(x,Y\right)$ as the (feature) resolved mutual information (rMI).

The mutual information for sequences $X\left(t\right)$ and $Y\left(t+\tau \right)$ at time (position) *t* with shift τ≥0 is defined as

(8) $$I\left(X\left(t\right),Y\left(t+\tau \right)\right)=\int_{X}\int_{Y}p\left(x\left(t\right),y\left(t+\tau \right)\right)\cdot \log\left(\frac{p\left(x\left(t\right),y\left(t+\tau \right)\right)}{p\left(x\left(t\right)\right)\cdot p\left(y\left(t+\tau \right)\right)}\right)dydx$$

which yields by setting $Y\left(t+\tau \right)=X\left(t+\tau \right)$

$$I\left(X\left(t\right),X\left(t+\tau \right)\right)=\int_{X}\int_{X}p\left(x\left(t\right),x\left(t+\tau \right)\right)\cdot \log\left(\frac{p\left(x\left(t\right),x\left(t+\tau \right)\right)}{p\left(x\left(t\right)\right)\cdot p\left(x\left(t+\tau \right)\right)}\right)dx\left(t+\tau \right)dx$$

as the auto mutual information at time/position *t* with shift (delay) τ [77,78]. If $p\left(x\left(t\right)\right)$ is independent from *t*, only the joint probability $p\left(x\left(t\right),x\left(t+\tau \right)\right)$ remains *t*-dependent or, more precisely, it becomes dependent only on the shift τ such that we simply write $p\left(x,x\left(\tau \right)\right)$ for this. Thus, the auto mutual information in dependence on the shift τ is obtained as

(9) $$I\left(X,\tau \right)=\int_{X}\int_{X}p\left(x,x\left(\tau \right)\right)\cdot \log\left(\frac{p\left(x,x\left(\tau \right)\right)}{p\left(x\right)\cdot p\left(x\left(\tau \right)\right)}\right)dx\left(\tau \right)dx$$

as an information theoretic analogous to the auto-correlation function. In [50,79], this shift-dependent auto mutual information is denoted as the mutual information function (MIF) $F\left(X,\tau \right)=I\left(X,\tau \right)$. Adapting the rMI from Equation (7) to the auto mutual information $I\left(X,\tau \right)$ results in the function

(10) $$\begin{array}{ccc} F\left(x,\tau \right) & = & \int_{X}p\left(x,x\left(\tau \right)\right)\cdot \log\left(\frac{p\left(x,x\left(\tau \right)\right)}{p\left(x\right)\cdot p\left(x\left(\tau \right)\right)}\right)dx\left(\tau \right)\\ & = & \int_{X}p\left(x,x\left(\tau \right)\right)\cdot \log\left(\frac{p\left(x,x\left(\tau \right)\right)}{p\left(x\left(\tau \right)\right)}\right)dx\left(\tau \right)-p\left(x\right)\cdot \log\left(p\left(x\right)\right) \end{array}$$

which can be seen as a quantity characterizing the inherent correlations of the sequence values $x\left(t\right)$. We denote $F\left(x,\tau \right)$ as the (feature) resolved mutual information function (rMIF), which trivially fulfills

(11) $$I\left(X,\tau \right)=\int_{X}F\left(x,\tau \right)dx$$

according to its definition. Note, more precisely would be the notation F(X,x,τ). We drop the dependency on *X* for better readability. For (finite) discrete distributions, it becomes simply a matrix F and $I\left(X,\tau \right)$ constitutes a vector. Hence, we can compare those vectors in terms of respective norms, e.g., by the Euclidean norm for vectors or the corresponding Frobenius norm for matrices [80,81].

### 2.2. Rényi α-Entropy and Related Mutual Information Functions

The Rényi-entropy

(12) $$H_{\alpha }^{R}\left(X\right)=\frac{1}{1-\alpha }\log\left(\int_{X}{\left(p\left(x\right)\right)}^{\alpha }dx\right)$$

is a generalization of the Shannon-entropy, where α>0 and α≠1 is a parameter [62]. Depending on the context, it is also denoted as α-entropy. In the limit α→1, the Shannon entropy is obtained. The corresponding Rényi-divergence is

(13) $$D_{\alpha }^{R}\left(p\left(x\right)\|p\left(y\right)\right)=\frac{1}{\alpha -1}\log\left(\int_{X}\int_{Y}\frac{{\left(p\left(x\right)\right)}^{\alpha }}{{\left(p\left(y\right)\right)}^{\alpha -1}}dydx\right)$$

with the limit $\lim_{\alpha \to 1}D_{\alpha }^{R}\left(p\left(x\right)\|p\left(y\right)\right)=D_{KL}\left(p\left(x\right)\|p\left(y\right)\right)$ being valid, such that the α-dependent Rényi-mutual-information (RMI) is defined as

(14) $$I_{\alpha }^{R}\left(X,Y\right)=D_{\alpha }^{R}\left(p\left(x,y\right)\|p\left(x\right)\cdot p\left(y\right)\right)$$

analogous to the Shannon case (3). This mutual information is widely applied in data analysis and pattern recognition as well as in information theoretic machine learning [82,83,84,85,86,87,88,89,90]. Unfortunately, a relation comparable to (6) does not hold, i.e.,

$$I_{\alpha }^{R}\left(X,Y\right)\neq H_{\alpha }^{R}\left(X\right)+H_{\alpha }^{R}\left(Y\right)-H_{\alpha }^{R}\left(X,Y\right)$$

is generally valid. This problem arises from the difficulty to define a conditional Rényi entropy to be consistent with the setting in the Shannon case [91,92,93]. Several variants are known [94,95]. The Jizba–Arimitsu conditional Rényi-entropy $H_{\alpha }^{\mathrm{JA}}\left(X|Y\right)$ defined as

(15) $$H_{\alpha }^{\mathrm{JA}}\left(X|Y\right)=H_{\alpha }\left(X,Y\right)-H_{\alpha }\left(Y\right)$$

fulfills the chain rule by definition [96]. Obviously, $H_{\alpha }^{\mathrm{JA}}\left(X|Y\right)$ can be interpreted as an extension of the conditional Shannon entropy $H\left(X|Y\right)$ because the definition (15) precisely coincides with Shannons chain rule (5). The resulting mutual entropy

(16) $$M_{\alpha }^{R}\left(X,Y\right)=H_{\alpha }\left(X\right)+H_{\alpha }^{\mathrm{JA}}\left(X|Y\right)$$

is consistent with (4) and preserves the symmetry [97]. However, it may violate the non-negativity as well as $I_{\alpha }^{R}\left(X,Y\right)\neq M_{\alpha }^{R}\left(X,Y\right)$ is valid. For further variants, we refer to [95].

Analogous to the resolved mutual information $F\left(x,Y\right)$ in the Shannon case from Equation (10), we denote

(17) $$F_{\alpha }^{R}\left(x,Y\right)=\int_{Y}\frac{{\left(p\left(x,y\right)\right)}^{\alpha }}{{\left(p\left(x\right)\right)}^{\alpha -1}\cdot {\left(p\left(y\right)\right)}^{\alpha -1}}dy$$

as the α-scaled (feature) resolved Rényi mutual information (rRMI). Obviously,

$$I_{\alpha }^{R}\left(X,Y\right)=\frac{1}{\alpha -1}\log\left(\int_{X}F_{\alpha }^{R}\left(x,Y\right)dx\right)$$

holds. The Rényi variant of the cross mutual information for sequences $X\left(t\right)$ and $Y\left(t+\tau \right)$ at time *t* with shift τ≥0 is defined as

(18) $$I_{\alpha }^{R}\left(X\left(t\right),Y\left(t+\tau \right)\right)=\frac{1}{\alpha -1}\log\left(\int_{X}\int_{Y}\frac{{\left(p\left(x\left(t\right),y\left(t+\tau \right)\right)\right)}^{\alpha }}{{\left(p\left(x\left(t\right)\right)\right)}^{\alpha -1}\cdot {\left(p\left(y\left(t+\tau \right)\right)\right)}^{\alpha -1}}dy\left(t+\tau \right)dx\left(t\right)\right)$$

which gives by setting $Y\left(t+\tau \right)=X\left(t+\tau \right)$

$$I_{\alpha }^{R}\left(X\left(t\right),X\left(t+\tau \right)\right)=\frac{1}{\alpha -1}\log\left(\int_{X}\int_{X}\frac{{\left(p\left(x\left(t\right),x\left(t+\tau \right)\right)\right)}^{\alpha }}{{\left(p\left(x\left(t\right)\right)\right)}^{\alpha -1}\cdot {\left(p\left(x\left(t+\tau \right)\right)\right)}^{\alpha -1}}dx\left(t+\tau \right)dx\left(t\right)\right)$$

as the Rényi variant of the auto mutual information at time *t* with shift (delay) τ. Again, if $p\left(x\left(t\right)\right)$ is independent from *t*, only the joint probability $p\left(x\left(t\right),x\left(t+\tau \right)\right)$ remains *t*-dependent such that it becomes dependent only on the shift τ and we simply write $p\left(x,x\left(\tau \right)\right)$ for this. Hence, the Rényi auto mutual information in dependence on the shift τ is obtained as

(19) $$I_{\alpha }^{R}\left(X,\tau \right)=\frac{1}{\alpha -1}\log\left(F_{\alpha }^{R}\left(X,\tau \right)\right)$$

with

(20) $$F_{\alpha }^{R}\left(X,\tau \right)=\int_{X}\int_{X}\frac{{\left(p\left(x,x\left(\tau \right)\right)\right)}^{\alpha }}{{\left(p\left(x\right)\right)}^{\alpha -1}\cdot {\left(p\left(x\left(\tau \right)\right)\right)}^{\alpha -1}}dx\left(\tau \right)dx$$

denoted as the Rényi variant of, or α-scaled Rényi mutual information function (RMIF). Accordingly, the α-scaled resolved version of the RMIF is

(21) $$F_{\alpha }^{R}\left(x,\tau \right)=\int_{X}\frac{{\left(p\left(x,x\left(\tau \right)\right)\right)}^{\alpha }}{{\left(p\left(x\right)\right)}^{\alpha -1}\cdot {\left(p\left(x\left(\tau \right)\right)\right)}^{\alpha -1}}dx\left(\tau \right)$$

describing again the inherent correlations of the sequence and, hence, can serve as a characterizing quantity of the sequence. Accordingly, we denote the function $F_{\alpha }^{R}\left(x,\tau \right)$ as the α-scaled resolved Rényi mutual information function (rRMIF) for Rényi entropies. Obviously,

(22) $$I_{\alpha }^{R}\left(X,\tau \right)=\frac{1}{\alpha -1}\log\left(\int_{X}F_{\alpha }^{R}\left(x,\tau \right)dx\right)$$

is valid analogous to Equation (11).

### 2.3. Tsallis α-Entropy and Related Mutual Information Functions

Recently, the Tsallis mutual information came into the focus for studying long range correlations in symbol sequences [70]. It is related to the Tsallis α-entropy

(23) $$H_{\alpha }^{T}\left(X\right)=\frac{1}{\alpha -1}\left(1-\int_{X}{\left(p\left(x\right)\right)}^{\alpha }dx\right)$$

which becomes in the limit α→1 the Shannon entropy $H\left(X\right)$. It was first introduced by Havrda and Charvát in 1967 [98] and later rediscovered by Tsallis [64]. It is related to the Rényi α-entropy $H_{\alpha }^{R}\left(X\right)$ by

$$H_{\alpha }^{R}\left(X\right)=\frac{\log\left(1-\left(1-\alpha \right)H_{\alpha }^{T}\left(X\right)\right)}{1-\alpha }$$

as stated in [65]. The Tsallis-divergence is given by

(24) $$D_{\alpha }^{T}\left(p\left(x\right)\|p\left(y\right)\right)=\frac{1}{\alpha -1}\left(1-\int_{X}\int_{Y}\frac{{\left(p\left(x\right)\right)}^{\alpha }}{{\left(p\left(y\right)\right)}^{\alpha -1}}dydx\right)$$

as explained in [64,74]. Using the same procedure as for the Shannon case (3), we obtain

(25) $$I_{\alpha }^{T}\left(X,Y\right)=D_{\alpha }^{T}\left(p\left(x,y\right)\|p\left(x\right)\cdot p\left(y\right)\right)$$

for the α-dependent Tsallis mutual information (TMI) [99]. As for the Rényi mutual information, the inequality

$$I_{\alpha }^{T}\left(X,Y\right)\neq H_{\alpha }^{T}\left(X\right)+H_{\alpha }^{T}\left(Y\right)-H_{\alpha }^{T}\left(X,Y\right)$$

is generally valid except the case α=1 being the Shannon case. It is symmetric and always non-negative but not consistent with the conditional Tsallis entropy

(26) $$H_{\alpha }^{T}\left(X|Y\right)=\frac{H_{\alpha }^{T}\left(X,Y\right)-H_{\alpha }^{T}\left(Y\right)}{1+\left(1-\alpha \right)\cdot H_{\alpha }^{T}\left(Y\right)}$$

as explained in [65]. To avoid these and other difficulties, finally, the Tsallis α-entropy based mutual entropy (information) is suggested to be

$$\begin{array}{ccc} M_{\alpha }^{T}\left(X,Y\right) & = & \frac{H_{\alpha }^{T}\left(X\right)+H_{\alpha }^{T}\left(Y\right)-H_{\alpha }^{T}\left(X,Y\right)+\left(1-\alpha \right)H_{\alpha }^{T}\left(X\right)H_{\alpha }^{T}\left(Y\right)}{1+\left(1-\alpha \right)H_{\alpha }^{T}\left(X\right)} \end{array}$$

as proposed in [65]. However, the inequality $M_{\alpha }^{T}\left(X,Y\right)\neq I_{\alpha }^{T}\left(X,Y\right)$ holds.

As for the Shannon and the Rényi variants of the mutual information, we consider a resolved Tsallis mutual information (rTMI)

(27) $$F_{\alpha }^{T}\left(x,Y\right)=\int_{Y}\frac{{\left(p\left(x,y\right)\right)}^{\alpha }}{{\left(p\left(x\right)\cdot p\left(y\right)\right)}^{\alpha -1}}dy$$

such that

(28) $$I_{\alpha }^{T}\left(X,Y\right)=\frac{1}{\alpha -1}\left(1-\int_{X}F_{\alpha }^{T}\left(x,Y\right)dx\right)$$

holds. For the auto mutual information with shift τ, we get

(29) $$I_{\alpha }^{T}\left(X,\tau \right)=\frac{1}{\alpha -1}\left(1-F_{\alpha }^{T}\left(X,\tau \right)\right)$$

with

(30) $$F_{\alpha }^{T}\left(X,\tau \right)=\int_{X}F_{\alpha }^{T}\left(x,\tau \right)dx$$

as the Tsallis mutual information function (TMIF) by the same arguments as before with

(31) $$F_{\alpha }^{T}\left(x,\tau \right)=\int_{X}\frac{{\left(p\left(x,x\left(\tau \right)\right)\right)}^{\alpha }}{{\left(p\left(x\right)\cdot p\left(x\left(\tau \right)\right)\right)}^{\alpha -1}}dx\left(\tau \right)$$

denoted as the α-scaled resolved Tsallis mutual information function (rTMIF) for Tsallis entropies. In bioinformatics context, it can be seen as a α-scaled object dependent average Tsallis mutual information profile.

Comparing TMIF and RMIF as well as rTMIF and rRMIF, we can obviously state the equalities $F_{\alpha }^{R}\left(X,\tau \right)=F_{\alpha }^{T}\left(X,\tau \right)$ and $F_{\alpha }^{R}\left(x,\tau \right)=F_{\alpha }^{T}\left(x,\tau \right)$.

All quantities relevant for the later data analysis are summarized and adapted for biomolecular sequences in Table 2.

## 3. Interpretable Classification Learning by Learning Vector Quantization

Learning vector quantization (LVQ) as introduced by T. Kohonen is a neural network approach for classification trained by Hebbian competitive learning to achieve an approximation of a Bayes classifier model [100,101]. It is based on the intuitive nearest prototype principle, i.e., prototype vectors are distributed in the data space during the learning phase to detect the data class distribution. In the recall phase, a data point is assigned to that class the nearest prototype is referencing based on a given data dissimilarity. It is known as a robust variant of the nearest neighbor principle [102]. In this way, LVQ is easy to interpret [27].

Particularly, LVQ supposes data vectors $x\in X={\left\{x_{k}\right\}}_{k=1}^{K}\subseteq R^{n}$ together with class labels $c\left(x\right)\in C=\left\{1,\ldots ,C\right\}$ for training [100]. Furthermore, the LVQ-model requires prototype vectors $w_{j}\in W={\left\{w_{k}\right\}}_{k=1}^{N}\subset R^{n}$ with class labels $c\left(w_{j}\right)$ such that each class of C is represented by at least one prototype. As already mentioned, a new data vector is assigned to a class by means of the nearest prototype principle

$$x\mapsto c\left(w^{*}\right)\;\mathrm{with}\;w^{*}=\underset{w_{j}\in W}{\mathrm{argmin}}\left(d\left(x,w_{j}\right)\right)$$

where $w^{*}$ is denoted as the winning prototype for the input x with respect to *W*. Here, *d* is a predefined dissimilarity measure in $R^{n}$ frequently chosen as the (squared) Euclidean distance. According to [103], prototype learning in GLVQ can be realized as a stochastic gradient descent learning (SGDL) for the prototype set W. The respective cost function

$$E=\sum_{x\in X}E\left(x,W\right)$$

approximates the overall classification error for the training set X by local errors

$$E\left(x,W\right)=f\left(\mu \left(x,W\right)\right)$$

taking into account the classifier function

$$\mu \left(x,W\right)=\frac{d\left(x,w^{+}\right)-d\left(x,w^{-}\right)}{d\left(x,w^{+}\right)+d\left(x,w^{-}\right)}$$

such that $\mu \left(x,W\right)\in \left[-1,1\right]$ is valid and *f* is a monotonically increasing sigmoid squashing function. Here, $w^{+}$ is the closest prototype to x with a correct label, whereas $w^{-}$ is the closest prototype with incorrect label, i.e.,

(32) $$\begin{array}{c} w^{+}=\underset{\begin{array}{c} w_{j}\in W\\ c\left(x\right)=c\left(w_{j}\right) \end{array}}{\mathrm{argmin}}d\left(x,w_{j}\right)\;\mathrm{and}\;w^{-}=\underset{\begin{array}{c} w_{j}\in W\\ c\left(x\right)\neq c\left(w_{j}\right) \end{array}}{\mathrm{argmin}}d\left(x,w_{j}\right) \end{array}$$

such that $\mu \left(x,W\right)<0$ holds in case of a correct classification.

The SGDL-step for a given input x is

$$\Delta w^{\pm }\propto \frac{\partial E\left(x,W\right)}{\partial w^{\pm }}$$

realizing an attraction scheme (vector shift) for $w^{+}$ towards x in case of the (squared) Euclidean distance as dissimilarity *d*, whereas $w^{-}$ is repelled from x. This variant of LVQ is known as generalized LVQ (GLVQ, [103]).

The interpretability and the power of the GLVQ can be improved taking the dissimilarity *d* as

(33) $$d_{\Omega }\left(x,w\right)={\left(\Omega \left(x-w\right)\right)}^{2}$$

where $\Omega \in R^{m\times n}$ is a mapping matrix with m≤n. This mapping matrix is also the subject of adaptation during learning with

$$\Delta \Omega_{ij}\propto \frac{\partial E\left(x,W\right)}{\partial \Omega_{ij}}$$

realizing the SGDL-step for a given input x. This approach is known as the generalized matrix LVQ (GMLVQ) [72]. In case of m<n, it is the limited rank GMLVQ (LiRaM-LVQ) [104].

The resulting matrix $\Lambda =\Omega^{T}\Omega$ is denoted as classification correlation matrix (CCM) [105]. The matrix entries $\Lambda_{ij}$ reflect after training those correlations between the $i^{th}$ and $j^{th}$ data features, which contribute to a class discrimination. More specifically, if $\left|\Lambda_{ij}\right|\gg 0$ is valid, the respective correlation of the features is important to separate the classes, whereas $\left|\Lambda_{ij}\right|\approx 0$ indicates that either the correlation between the $i^{th}$ and $j^{th}$ data feature does not improve the classification or that this correlation information is already contained in another significant correlation. The vector $\lambda ={\left(\lambda_{1},\ldots ,\lambda_{n}\right)}^{T}$ with

$$\lambda_{i}=\Lambda_{ii}$$

being the non-negative diagonal elements of the classification correlation matrix is denoted as classification relevance profile (CRP) of the features [106]. It describes the relevance of the features for class discrimination with an analog interpretation as for $\left|\Lambda_{ij}\right|$. The classification influence profile (CIP), defined as $\kappa ={\left(\kappa_{1},\ldots ,\kappa_{n}\right)}^{T}$ with

$$\kappa_{i}=\sum_{j}\left|\Lambda_{ij}\right|$$

provides the importance of the $i^{th}$ data feature in combination with all other features for the separation of the data set. Both profiles, as well as the classification correlation matrix, provide additional information beyond the pure classification performance and, hence, contribute to a high interpretability of the classification model [107].

Moreover, all mentioned GLVQ variants are robust classification learning models maximizing the hypothesis margin for most appropriate class separation [73,108].

## 4. Applications of Mutual Information Functions for Sequence Classification

In the following, we apply the described information theoretic quantities as characterizing features for biomolecular sequences. Particularly, we use the introduced variants of mutual information functions summarized in Table 2 and natural vectors as feature generators. Their performance is evaluated in combination with the LiRaM-LVQ for three biological classification tasks.

### 4.1. Data Sets

The chosen data sets summarized in Table 1 are representatives of biological applications facing the common challenges of varying sequence lengths and containing ambiguous characters (see Section 4.2.3).

#### 4.1.1. Quadruplex Detection

This data set consists of 368 nucleotide sequences that were experimentally validated to either build or not build a G-quadruplex during folding. Quadruplexes are structural (3D) motifs of one or more nucleic acid strands consisting of at least two stacked tetrads. These are characterized by the planar arrangement of four nucleotides, each of which forms non-canonical bonds (base pairing schemes other than Watson-Crick) with two of the other nucleotides. If all tetrad-forming nucleotides are guanine, it is also denoted as the G-quadruplex, or G4. The utilized data are equivalent to that published by [109] without the random sequences (background sequences assumed to be non-G4). The data source is the G4RNA database [110].

#### 4.1.2. lncRNA vs. mRNA

For the next task, we used a data set containing 10,000 human long non-coding RNA (lncRNA) sequences and 10,000 protein-coding transcripts (mRNA). lncRNA are transcripts that do not encode proteins, i.e., they are not translated, but play a role in gene regulation. Their typical length of more than 200 nucleotides (nt) delineates them from small non-coding RNA such as miRNAs or snoRNAs and similarities in sequence structure compared to mRNA make their differentiation challenging [111]. The data set was generated analogous to [111]: The data were retrieved from the GENCODE database [112] in the latest version v.38 at the time of access (11 August 2021). Data preprocessing comprising filtering of sequences with length 250–3000 nt and random selection of 10,000 sequences per class was applied. In contrast to [111], we decided to use the same interval for both classes in order to not prone our classifier to use the sequence length as class discriminating property.

#### 4.1.3. COVID Types

As a third data set, we took 156 coronavirus sequences from human hosts of the types A, B, and C, implicitly coding the evolution in time of the virus. The SARS-CoV-2 sequence data source is the GISAID (Global Initiative on Sharing Avian Influenza Data) coronavirus repository from 4 March 2020 with types derived from a phylogenetic network analysis in [113]. Type A is most similar to the bat virus, type B evolves from A by a non-synonymous and a synonymous mutation (evolutionary substitutions that do or do not modify the resulting amino acid sequence, respectively) and type C is characterized by a further non-synonymous mutation.

### 4.2. Feature Generation

In the following, we introduce the concept of natural vectors and provide a description of how to generate feature vectors from the information theoretical quantities MIF and rMIF introduced in Section 2 for machine learning applications. Both feature generators are sequence length independent and capable of handling ambiguous characters in biological data as covered in more detail in Section 4.2.3.

#### 4.2.1. Natural Vectors

Natural vectors (NV) in biomolecular context accumulate statistical descriptors concerning the distribution of nucleotide positions within a sequence $s=[s_{1}\ldots s_{n}]$ over the alphabet A={A,C,G,T}. They were generalized in [47] from [114]. Natural vectors are known to be characteristic fingerprints for biomolecular sequences reflecting statistical and, hence, information theoretic properties. Therefore, we consider them as baseline for comparison with mutual information functions.

To define NV accurately, let $n_{k}=\sum_{i=1}^{n}w_{k}\left(s_{i}\right)$ be the absolute frequency of nucleotide *k* in *s*, given that $w_{k}\left(s_{i}\right)\in \left\{0,1\right\}$ indicates the absence (0) or presence (1) of *k* at sequence position $s_{i}$. Furthermore, let $\mu_{k}=\sum_{i=1}^{n}i\cdot \frac{w_{k}\left(s_{i}\right)}{n_{k}}$ denote the mean sequence position of nucleotide *k* and $D_{k}^{j}=\sum_{i=1}^{n}\frac{{\left(i-\mu_{k}\right)}^{j}w_{k}\left(s_{i}\right)}{n_{k}^{j-1}n^{j-1}}$ be the normalized central moment of order *j*. Then, the natural vector is defined as   

(34) $$x=(n_{A},\mu_{A},D_{A}^{2},\ldots ,D_{A}^{n_{A}},n_{C},\mu_{C},D_{C}^{2},\ldots ,D_{C}^{n_{C}},n_{G},\mu_{G},D_{G}^{2},\ldots ,D_{G}^{n_{G}},n_{T},\mu_{T},D_{T}^{2},\ldots ,D_{T}^{n_{T}})$$

   Obviously, $\mu_{k}=D_{k}^{1}$ is valid and one can take just $n_{k}=D_{k}^{0}$ in terms of the statistical moments. Furthermore, it was stated that this setting guarantees a unique coding of the molecular sequences [47]. In practical applications, the maximum order $j_{\max}$ of moments to be calculated is fixed equally in dependence of the data set for all nucleotides to achieve equal-length vectors for all considered sequences. Hence, the data dimension becomes $n=4\cdot (j_{\max}+1)$.

In the experiments, we determined an optimal setting of $j_{\max}$ under consideration of the sequence length via grid search. Therefore, we evaluated $j_{\max}\in \left\{2,3,4\right\}$, {2,…,15} and {2,…,15} for the Quadruplex detection, lncRNA vs. mRNA and COVID types data set, respectively. We directly take x from Equation (34) as input (feature vector) for the LVQ model. The maximum order 15 for $j_{\max}$ was taken as an upper bound because higher moments were numerically vanishing for the used data sets.

#### 4.2.2. Mutual Information Functions

In case of mutual information functions, the feature vector $x={\left(x_{1},\ldots ,x_{\tau_{\max}}\right)}^{T}$ is generated from a sequence *X* by setting $x_{\tau }=F\left(X,\tau \right)$ or $x_{\tau }=F_{\alpha }^{R}\left(X,\tau \right)$ for Shannon and Rényi, respectively. The maximum distance between pairs of nucleotides considered in the sequence is $\tau_{\max}$.

For the resolved mutual information functions, we take

$$x={\left(x_{1}^{A},\ldots ,x_{\tau_{\max}}^{A}\;\;x_{1}^{C},\ldots ,x_{\tau_{\max}}^{C}\;\;x_{1}^{G},\ldots ,x_{\tau_{\max}}^{G}\;\;x_{1}^{T},\ldots ,x_{\tau_{\max}}^{T}\right)}^{T}$$

with $x_{\tau }^{k}=F\left(k,\tau \right)$ or $x_{\tau }^{k}=F_{\alpha }^{R}\left(k,\tau \right)$, where k∈A={A,C,G,T}.

Table 2 summarizes the applied mutual information functions for the Shannon and Rényi case.

In the literature on MIF, there is disagreement on how to calculate the marginal probabilities of the nucleotides: one camp propagates a symmetric version, i.e., p(x) denotes the relative frequency of a nucleotide *x* in a sequence [52,54,56], while the other distinguishes the frequencies of the nucleotides at the positions *x* depending on x(τ), i.e., $p\left(x\right)=\sum_{x(\tau )}p\left(\left(x,x(\tau )\right)|x\right)$ and $p\left(x(\tau )\right)=\sum_{x}p\left(\left(x,x(\tau )\right)|x\left(\tau \right)\right)$ [57,58,59]. We used the latter (non-symmetric) version, since biological sequences have a chemically reasonable reading direction, such that a nucleotide’s neighbor is determined in the 3’ direction.

An optimal setting of the hyper-parameter $\tau_{\max}$ was obtained under consideration of the sequence length via grid search. We evaluated $\tau_{\max}\in \left\{2,\ldots ,8\right\}$, {10,25,50,100} and {5,10,50,100} for the Quadruplex detection, lncRNA vs. mRNA and COVID types data set, respectively.

The α-value for the Rényi variants was set to α=2 as usual. This choice leads to low computational costs and provides numerical stability [82].

#### 4.2.3. Handling of Ambiguous Characters

Ambiguous characters are introduced by the IUPAC (International Union of Pure and Applied Chemistry) degenerate base notation [115]. Thereby, the notation ambiguous refers to the concept that a single character from the alphabet extension

E={R,Y,M,K,S,W,H,B,V,D,N} represents more than one nucleotide, present in data to describe incompletely specified bases or uncertainty of them [115]. For instance *R* denotes either *A* or *G*, the ambiguous character *H* stands for either *A*, *C*, or *T*, whereas *N* codes for all four possible nucleotides.

In order to make the feature generators cope with these representations, the weights $0\leq w_{k}\left(s_{i}\right)\leq 1$ now code the probability for, and not just the presence (1) or absence (0) of, a nucleotide at one specific sequence position, i.e.,

(35) $$w_{A}\left(s_{i}\right)=\left\{\begin{array}{cc} 1 & if\;s_{i}=A\\ 0 & if\;s_{i}=C,G,T,Y,K,S\;\mathrm{or}\;B\\ \frac{1}{2} & if\;s_{i}=R,M\;\mathrm{or}\;W\\ \frac{1}{3} & if\;s_{i}=H,V\;\mathrm{or}\;D\\ \frac{1}{4} & if\;s_{i}=N \end{array}\right.\;.\;$$

In [116], natural vectors were expanded to handle this extended alphabet. We designed a solution for the MIF variants analogously.

### 4.3. Classification

Following all the feature extractors mentioned above, we have applied a Z-score normalization in order to make the individual features comparable. Classification was then done using the LiRaM-LVQ implementation from the Python toolbox prototorch in 3-fold cross validation. In all cases, the prototypes for learning were initialized as randomly selected data points and the learning rate was set to 0.01 in all cases. The mapping dimension *m* was set to 10 independent of data set or feature set. The choice of the number of prototypes was data set depending: for Quadruplex detection and COVID types, we took only one prototype per class. For the lncRNA vs. mRNA data set, the grid search for optimal setting resulted in 50 prototypes per class as balance between complexity of the model and performance.

## 5. Results and Discussion

### 5.1. Classification Performance

Table 3 displays the achieved test accuracies by LiRaM-LVQ in combination with the optimal parameter setting of $j_{\max}$ and $\tau_{\max}$ for the NV and MIF variant feature extractors, respectively.

Considering these results in Table 3, we see that rMIF outperforms the MIF variants as well as the models which use NV for feature generations for all three data sets. Furthermore, the developed Rényi variant shows in the Quadruplex detection example for rMIF significantly better results compared to the Shannon counterpart. However, for the second data set, the performance of rMIF depends on the choice of $\tau_{\max}$. In general, it can be said that for long sequences $\tau_{\max}$ need to be chosen adequately if long range correlations are to be considered as well.

For deeper investigation of these results and to show the capabilities of the applied LiRaM-LVQ classifier, we will consider the CCM and CIP. Furthermore, visualizations of the mean MIF and rMIF per class and data set are considered for deeper understanding of the generated features and their potential differences between classes. In order to not overload the reader, we will restrict a more in-depth interpretation and discussion to one of the data sets, the quadruplex detection challenge.

It should be noted that a feature generation procedure based on pure statistics might achieve comparable or even better results. For this reason, it is not surprising that the statistical feature extractor Bag of words [117,118] has been successful in related works on the data sets mentioned: 92.8% AUC were achieved for the quadruplex data in combination with a simple neural network [109], an accuracy of 98.7% was described in [111] for the lncRNA vs. mRNA data by use of a convolutional neural network and 97.4% accuracy were obtained in [119] for COVID type detection using GMLVQ. However, the focus of this paper is on the investigation and further development of information theoretical methods and their suitability for sequence analyses in computational biology.

### 5.2. Visualization of MIF Variants

**Figure 1:**
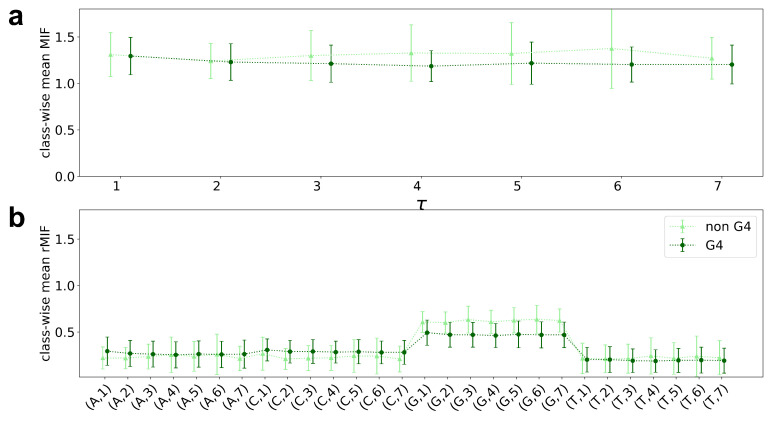
Data insights, i.e., class-wise mean and standard deviation of the MIF variants for the quadruplex data set (G4 and non-G4 forming sequences). (**a**) Rényi MIF; (**b**) Rényi rMIF.

A closer look at the class-wise averaged MIF variant profiles in Figure 1 allows for assessing the methods behavior on the quadruplex data set. The plotted means suggest a clear class delineation, while the standard deviation adds depth/difficulty to the problem. All profiles are plotted prior to Z-score normalization, but with a slight vertical shift between the classes for better visual perception.

Comparison of the MIF and rMIF clearly shows a more accurate resolution of the information for rMIF, not only in terms of inter-sequential distances, but also in terms of individual nucleotides. Obviously, the sum of the four F(x,τ) with x∈{A,C,G,T} yields F(X,τ). The features with respect to G-nucleotides stand out in particular.

### 5.3. Interpretation of CCM and CIP of the Trained LiRaM-LVQ Model

The resulting CCM of the trained LiRaM-LVQ model gives domain experts, here biologists, immediate assistance to evaluate whether the classifier works reasonably. Furthermore, it allows statements to be made about whether the classification decision is based on some data biases or artifacts that were not necessarily known during data generation [104]. This interpretation possibility of the LiRaM-LVQ model is a huge advantage in comparison to black box models [120] especially in biological issues: Together with meaningful data features, as given here, biologists can draw conclusions regarding expected biological and biochemical properties.

In the experiments, we verified that the CCM can serve as a basis for interpretation by repeating the classification process multiple times and analyzing whether the matrix is visually stable. If significant deviations had been seen, an interpretation would have been spurious. Each depicted CCM is the result of averaging the individual CCMs obtained form the three validation folds. Furthermore, we limit the visualization to the best hyper-parameter setting according to our grid search.

**Figure 2:**
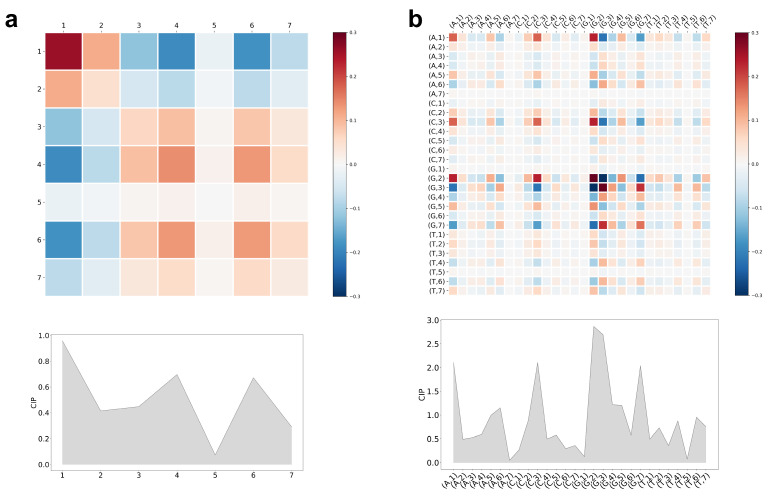
Classification insights, i.e., CCM and CIP of LiRaM-LVQ for the quadruplex data set. The color bars code the correlation values of the CCM. (**a**) Rényi MIF; (**b**) Rényi rMIF.

For the quadruplex data set, the best choice $\tau_{\max}=7$ gives a CCM with dimensionality 7×7 and 28×28 for the MIF and rMIF case, respectively. These quantities are visualized in Figure 2 giving insights into the classification decision of LiRaM-LVQ:

As can be seen from the CIP and from the CCMs’ main diagonal, the CRP, in Figure 2a, the MIF values for τ equals 1, 4, and 6 mainly influence the classifier’s decision to discriminate the classes of G-quadruplex (G4) and non-G4 forming sequences. Moreover, the CCM shows positive and negative correlations *between* the features. For example, features τ=4 and 6 are strongly positive and τ=1 and 4 are in strong negative correlation with each other. Thus, if τ=1 has a high value, it is important for the class discrimination, but only if τ=4 has a small value and vice versa. It is striking that the feature τ=5 does neither alone nor in combination with any other feature contribute to the differentiation for this learned model.

In Figure 2b, the CIP illustrates that eight features stand out with their influence on the class discrimination. Sorted by importance, these are: the information for (G,2), (G,3), (A,1), (C,3), (G,7), (G,4), (G,5), and (A,6). Taking the CCM into consideration, a high positive correlation between (A,1) and (G,2) as well as between (C,3) and (G,2) is obvious. Examples for high negative correlations would be the pairs of (G,2) and (G,3) or between (A,1) and (G,3). The clearly recognizable significance of Gs at different distances is biologically sound due to the general characterization of a G-quadruplex by a pattern of recurring guanines in the sequence as described in [121]. Insights like this would not have been possible with the standard MIF but only with our introduced resolved variant rMIF.

At first glance, one might claim an inconstancy between the high influence values in the CIPs for MIF and rMIF features. However, in the MIF calculation, there is an averaging of the information over the alphabet, such that the classifier can make use of more detailed information with rMIF. This means that the summation of the classification influence values for all four (x,τ) does not necessarily result in the influence value for the MIF for a specific τ and vice versa. As the individual nucleotides play a key role in the bioinformatics domain, there might be an essential information loss if an averaging procedure takes place as it is done for the simpler MIF.

Beside biological interpretations, these insights offer the possibility to adjust or rather fine-tune the classification model. For example, by taking just the seven most important rMIF features into account, we still obtain a performance of 77.1±0.7%. Hence, we could reduce the model complexity with moderate performance decrease.

To sum up, the LiRaM-LVQ classifier is transparent in the decision process as well as in the Hebbian learning process. Now, the expert can start to evaluate the results and either extract knowledge from the classifier or question the quality of the data/model if the results seem peculiar.

**Figure 3:**
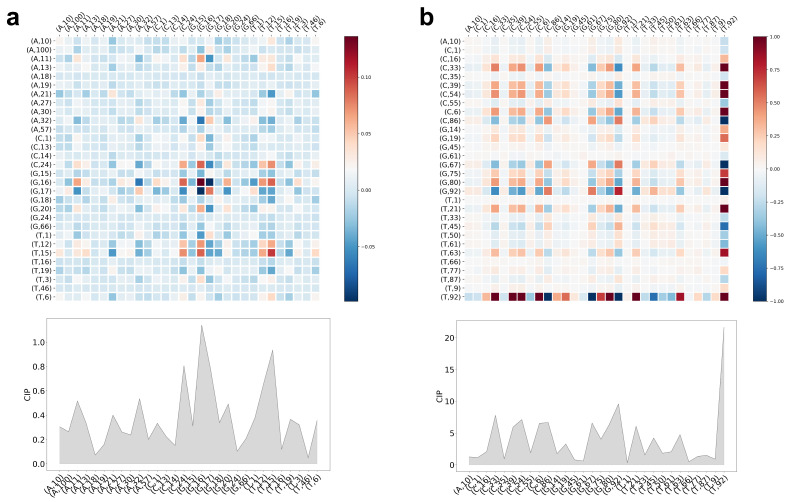
Classification insights, i.e., CCM and CIP of LiRaM-LVQ using the Shannon rMIF. The color bars code the correlation values of the CCM. (**a**) COVID data; (**b**) lncRNA vs. mRNA data.

For the sake of completeness, Figure 3 shows the CCM and CIP for the lncRNA vs. mRNA as well for the COVID type data set. The Shannon rMIF features were superior in these tasks. Our grid search resulted in high optimal values for $\tau_{\max}$ which is alright for pure performance evaluation but poses a problem in visually evaluating the CCMs and drawing conclusions. Therefore, we decided to take advantage of the same procedure described above: we identified the 30 most important/valuable features using the CIP, ran the classification procedure again using only these, and finally visualized both characteristics.

An in-depth analysis of the results including biological interpretation is up to the well-disposed reader.

## 6. Conclusions, Remarks, and Future Work

In this contribution, we propose information theoretic concepts and quantities to characterize spatial correlations in sequences. In particular, we introduced several variants of mutual information functions for Shannon, Rényi, and Tsallis information theoretic approaches. In particular, the resolved mutual information functions provide subtle information regarding the internal spatial correlation of the sequences.

These functions/quantities can be used as sequence signatures/fingerprints and thus for comparison in machine learning approaches. In particular, interpretable machine learning models can make use of this resolved information to achieve insights about the sequence class differences. As we have shown using our favored LiRaM-LVQ, detailed information can be extracted as an add-on to the pure classification model.

We see applications for sequence analysis in bioinformatics, especially in the context of alignment-free sequence comparison. Additionally, we remark that this concept can be extended to the analysis of more general categorical sequential data such as natural language texts or sheet music.

In the future work, we will extend this approach to further mutual information concepts related to other widely considered entropy measures and information theoretic quantities, e.g., the Cauchy–Schwarz-divergence [85], or more general α-, β- and γ-divergences with related mutual information concepts [74,91,122]. Further considerations could be a generalization to higher than two-body correlations as suggested in [123] or performing the calculation for sequences not 1 by 1 residue (position), but multiple residues [59].

Furthermore, we want to compare these methods with other feature generators taking statistical (spatial) correlation into account such as the return time distribution [124] known from stochastic modeling, DMk method [125] incorporating the occurrence, location, and order relation of *k*-mers, compression based methods with the underlying concept of minimum description length [126], methods based on domain transform, i.e., Fourier/Wavelet [127,128], DNA walks [45,129] and iterated function systems, e.g., chaos game representation or universal sequence maps [42,130].

However, interpretability should be kept always as a key feature when considering alternative models [25,131,132]. Interpretability increases the trustworthiness and hence the acceptance of models for the potential users [27]. Further extensions improving transparency of the decision and already known for GLVQ approaches are the incorporation of reject options for ambiguous decisions or outliers as well as the use of interpretable probabilistic classifiers [133,134,135].

## Figures and Tables

**Table 1:** Overview of the used data sets.

Data Set	Classes	Sequences	Per Class *	Mean Length	Std. Length
Quadruplex detection	2	368	175/193	62.1	43.7
lncRNA vs. mRNA	2	20,000	10,000 each	1197.3	710.8
COVID types	3	156	44/90/22	29,862.9	34.1

* Sample size per class.

**Table 2:** Overview of the computed mutual information functions for biomolecular sequences *X* from Section 2.1 and Section 2.2.

	MIF	rMIF
Shannon	$F\left(X,\tau \right)=\sum_{x\in X}F\left(x,\tau \right)$	$F\left(x,\tau \right)=\sum_{x(\tau )\in X}p\left(x,x(\tau )\right)\cdot \log\left(\frac{p\left(x,x(\tau )\right)}{p\left(x\right)\cdot p\left(x(\tau )\right)}\right)$
Rényi	$F_{\alpha }^{R}\left(X,\tau \right)=\sum_{x\in X}F_{\alpha }^{R}\left(x,\tau \right)$	$F_{\alpha }^{R}\left(x,\tau \right)=\sum_{x(\tau )\in X}\frac{p{\left(x,x(\tau )\right)}^{\alpha }}{{\left(p\left(x\right)\cdot p\left(x(\tau )\right)\right)}^{\alpha -1}}$

**Table 3:** Achieved test accuracies ± standard deviation by LiRaM-LVQ in percent and the respective parameter setting.

Data Set	NV	MIF		rMIF	
		Shannon	Rényi	Shannon	Rényi
Quadruplex detection	78.8±1.0 $j_{\max}=4$	68.9±1.2 $\tau_{\max}=7$	68.2±1.7 $\tau_{\max}=7$	77.4±1.3 $\tau_{\max}=8$	82.0±1.0 $\tau_{\max}=7$
lncRNA vs. mRNA	71.9±0.1 $j_{\max}=7$	75.4±0.2 $\tau_{\max}=100$	75.5±0.3 $\tau_{\max}=100$	81.4±0.1 $\tau_{\max}=100$	76.3±0.6 $\tau_{\max}=100$
COVID types	86.0±1.2 $j_{\max}=5$	98.1±0.6 $\tau_{\max}=50$	97.4±1.0 $\tau_{\max}=50$	99.7±0.3 $\tau_{\max}=50$	99.3±0.5 $\tau_{\max}=50$

## Data Availability

The data set for quadruplex detection is publicly available at https://academic.oup.com/bioinformatics/article/33/22/3532/4061281#supplementary-data, that for lncRNA vs. mRNA at https://www.gencodegenes.org/human/ (version v.38), and the accession numbers for the COVID type detection at https://www.springerprofessional.de/learning-vector-quantization-as-an-interpretable-classifier-for-/19111526?fulltextView=true (all accessed on 11 August 2021). The toolbox prototorch is publicly available at https://github.com/si-cim/prototorch and was used in version 0.2.0. The code for the NV and MIF variant calculation can be obtained from the authors upon request.

## Abbreviations

The following abbreviations are used in this manuscript:

AMI	Average Mutual Information
CCM	Classification Correlation Matrix
CIP	Classification Influence Profile
GISAID	Global Initiative on Sharing Avian Influenza Data
GLVQ	Generalized Matrix Learning Vector Quantization
IUPAC	International Union of Pure and Applied Chemistry
lncRNA	Long Non-Coding RNA
LiRaM-LVQ	Limited Rank Matrix Learning Vector Quantization
LVQ	Learning Vector Quantization
MIF	Mutual Information Function
mRNA	messenger RNA
NV	Natural Vectors
rMIF	resolved Mutual Information Function
RMIF	Rényi Mutual Information Function
rRMIF	resolved Rényi Mutual Information Function
rTMIF	resolved Tsallis Mutual Information Function
SGDL	Stochastic Gradient Descent Learning
TMIF	Tsallis Mutual Information Function
